# Association Between Chronotypes and Addiction Among Adults – A Systematic Review

**DOI:** 10.1192/bjo.2025.10230

**Published:** 2025-06-20

**Authors:** Manjula Simiyon, Parthasarathy Ramamurthy, Dheeraj Kattula, Steven Jones

**Affiliations:** 1 Cygnet Health Care, Mold, United Kingdom; 2 Pondicherry Institute of Medical Sciences, Pondicherry, India; 3 Christian Medical College, Vellore, India; 4 Chester Medical School, Chester, United Kingdom

## Abstract

**Aims:** Chronotype refers to the distribution of an individual’s diurnal preference, ranging from morning type (MT) to intermediate or neither type (NT) to evening type (ET). Chronotype manifests in various physiological functions and influences numerous physical and psychological activities. Addiction is one of the most prevalent mental health issues, with individual studies indicating that ETs are at a greater risk of developing addiction. Given the nonexistence of a systematic review addressing this topic, this study aimed to synthesize results that explored the relationship between chronotype and addictive disorders to define the at-risk population.

**Methods:** A search strategy was developed using the keywords 'chronotype', 'circadian preference', 'diurnal preference', 'morningness', 'eveningness', 'circadian rhythms’, and 'chronobiology'. And 'addiction', 'dependence', 'problematic use’ and 'abuse'. After registering the protocol with PROSPERO, a data extraction form was developed based on the strict inclusion and exclusion criteria for the selection of studies. Selected articles were screened and the data were extracted. The results of the 16 selected studies were synthesised.

**Results:** ET had a significant association with substance addiction in four out of six studies and all ten studies of behavioural addiction. One study showed no association with chronotype and one showed a significant association with MT.

**Conclusion:** ET has a higher risk of developing an addiction. Designing and conducting longitudinal cohort studies would be beneficial in delineating the bidirectional relationship and establishing causality if present. This would also pave the way for developing chronotherapies for addiction.

